# High‐Entropy Sulfides Catalyze Rate‐Determining Redox in Fast‐Charging Aqueous Zinc–Sulfur Batteries

**DOI:** 10.1002/anie.202503472

**Published:** 2025-05-13

**Authors:** Jiahao Liu, Han Wu, Chao Ye, Shi‐Zhang Qiao

**Affiliations:** ^1^ School of Chemical Engineering The University of Adelaide Adelaide SA 5005 Australia

**Keywords:** Fast‐charging batteries, High‐entropy catalysts, Rate‐determining step, Zinc–sulfur batteries

## Abstract

The sluggish kinetics of the solid–solid Zn–S redox process significantly hinders the practical energy density and lifespan of fast‐charging aqueous Zn–S batteries (AZSBs). Conventional low‐entropy catalysts suffer from poor stability, leading to leaching effects and water splitting during cycling. To overcome these limitations, we present a three‐step synthesis of high‐entropy sulfide (HES) nanorod catalysts to accelerate the rate‐determining step (RDS) in the Zn–S redox process. Operando synchrotron powder diffraction, operando synchrotron infrared reflectance microscopy, and operando Raman spectroscopy characterizations reveal that the HES catalysts improve sulfur utilization by accelerating the RDS conversion of ZnS_2_ to wurtzite ZnS. Furthermore, near‐edge X‐ray absorption fine structure and inductively coupled plasma mass spectrometry analyses demonstrate that the HES catalysts effectively suppress the leaching effect of transition metals and water splitting of the aqueous electrolyte, improving cycling stability. In contrast, utilizing medium‐ and low‐entropy catalysts results in the formation of by‐products, including S_5_^2−^, S_3_^2−^, and SO_3_^2−^ species. Consequently, the pouch cell with the HES catalysts delivers a high cathode energy density of 313 Wh kg^−1^ and high cycling stability over 400 cycles at 4 C with 0.06% capacity decay per cycle. This entropy‐driven catalytic strategy provides an effective approach for developing stable and fast‐charging aqueous metal–sulfur batteries.

## Graphical abstract

This work reveals how entropy regulates sulfur utilization and cycling stability in aqueous Zn–S batteries. High‐entropy (HES) catalysts enable optimized Zn–S redox pathways and structural integrity, achieving high sulfur utilization and stable cycling. In contrast, low‐ (LES) and medium‐entropy (MES) catalysts show inefficient reactions with by‐product formation and Mn/Co leaching, degrading performance. Practical validation demonstrates HES‐based pouch cells maintain exceptional stability over 400 cycles at 4 C with merely 0.06% capacity decay per cycle, establishing entropy engineering as a practical design strategy.

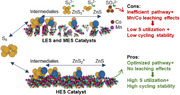

## Introduction

Aqueous zinc‐based batteries hold promise for fast‐charging energy storage applications owing to the fast ionic conductivity of aqueous electrolytes.^[^ ^1^, ^2^, ^3^ ^]^ When Zn anodes are coupled with high‐capacity cathode materials such as sulfur (1675 mAh g^−1^), the resultant aqueous Zn–S batteries (AZSBs) are a suitable candidate to meet the fast‐charging criteria (>4 C, equal to 6.7 A g^−1^ in AZSBs).^[^ ^4^ ^]^ However, the practical application of fast‐charging AZSBs is hindered by challenges such as slow solid–solid redox kinetics (S_8_‐ZnS_6_^*^‐ZnS_4_^*^‐ZnS_2_^*^‐ZnS), low electronic conductivity of sulfur‐containing species, and significant volumetric strain under fast‐charging conditions. Among these, the kinetic challenges of the solid–solid redox process are pivotal for achieving fast‐charging performance as they critically influence the practical sulfur utilization and battery lifespan.^[^ ^4^ ^]^ However, addressing these kinetic challenges remains difficult due to the insufficient understanding of the rate‐determining step (RDS) in this solid–solid Zn–S redox process.

Recent studies have aimed to identify and accelerate the RDS in the solid–solid Zn–S redox process. Theoretical computational studies by Zhang et al.^[^ ^5^ ^]^ suggested that the energy barrier of the conversions related to ZnS_2_* is the RDS. To accelerate it, transition‐metal sulfide‐, carbide‐, and nitride‐based catalysts have been designed and synthesized.^[^ ^5^ ^]^ However, the absence of advanced operando characterization techniques has impeded a comprehensive understanding of the RDS, therefore the structure–activity relationship of catalysts and their performance remains unclear. More importantly, conventional low‐entropy catalysts suffer from poor physical and chemical stability, resulting in a leaching effect and water splitting during charge–discharge cycles. Consequently, the resultant cycling stability of low‐entropy catalysts degrades significantly over long‐term cycling under fast‐charging conditions.^[^ ^6^ ^]^

High‐entropy materials, including alloys, oxides, and sulfides,^[^ ^7^ ^]^ have shown significant potential as multifunctional catalysts due to their unique high‐entropy and cocktail effects, in which the former enhances catalysts’ stability by promoting robust solid–solution formation, whereas the latter boosts catalytic performance via synergistic multielement interactions.^[^ ^8^ ^]^ These hold the potential to enhance sulfur utilization and improve cycling stability in fast‐charging AZSB systems.^[^ ^7^ ^]^ More importantly, by optimizing their components and structures, high‐entropy catalysts can be designed to suppress water splitting, particularly the oxygen evolution reaction (OER), a major challenge in their application to aqueous‐based batteries.^[^ ^8^, ^9^ ^]^ For instance, Hu et al.^[^ ^10^ ^]^ demonstrated this by using a high‐entropy oxide (Co, Ni, Fe, Mn, Cu)O*_x_*, where the unique high‐entropy structure effectively reduced electronic degeneracy and optimized the d band center, thereby suppressing OER and selectively enhancing the target redox reactions of the battery during prolonged cycling.

In this study, we reveal the pivotal structure–activity relationship, showing that the catalyst entropy fundamentally enhances sulfur utilization and cycling stability of the fast‐charging AZSBs. First, to explore the impact of catalyst entropy on sulfur utilization, operando synchrotron powder diffraction (PD), operando synchrotron infrared reflectance microscopy (IRM), and operando Raman spectroscopy analyses demonstrate that high‐entropy sulfide (HES) catalysts effectively accelerate the reversible RDS conversion of ZnS_2_^*^ to wurtzite ZnS by optimizing the reaction pathway. In contrast, by‐product formation occurs in low‐ and medium‐entropy systems due to their inferior control of the reaction pathway. In addition, in situ gas chromatography–mass spectrometry (in situ GCMS), near‐edge X‐ray absorption fine structure (NEXAFS), and inductively coupled plasma mass spectrometry (ICPMS) analyses demonstrate that HES catalysts can avoid the leaching effects and minimize water splitting. In contrast, low‐ and medium‐entropy catalysts exhibit severe Mn/Co leaching during cycling. As a result, the pouch cell with 50 mg sulfur loading and the HES catalysts delivers a high cathode energy density of 313 Wh kg^−1^ and high cycling stability over 400 cycles at 4 C with 0.06% capacity decay per cycle. This work establishes an entropy‐driven catalyst design strategy that accelerates the solid–solid redox process, suppresses by‐products, and significantly enhances the long‐term stability of fast‐charging aqueous batteries, thereby paving the way for developing next‐generation aqueous metal–sulfur batteries.

## Results and Discussion

### Three‐Step Synthesis of HES

**Figure 1:**
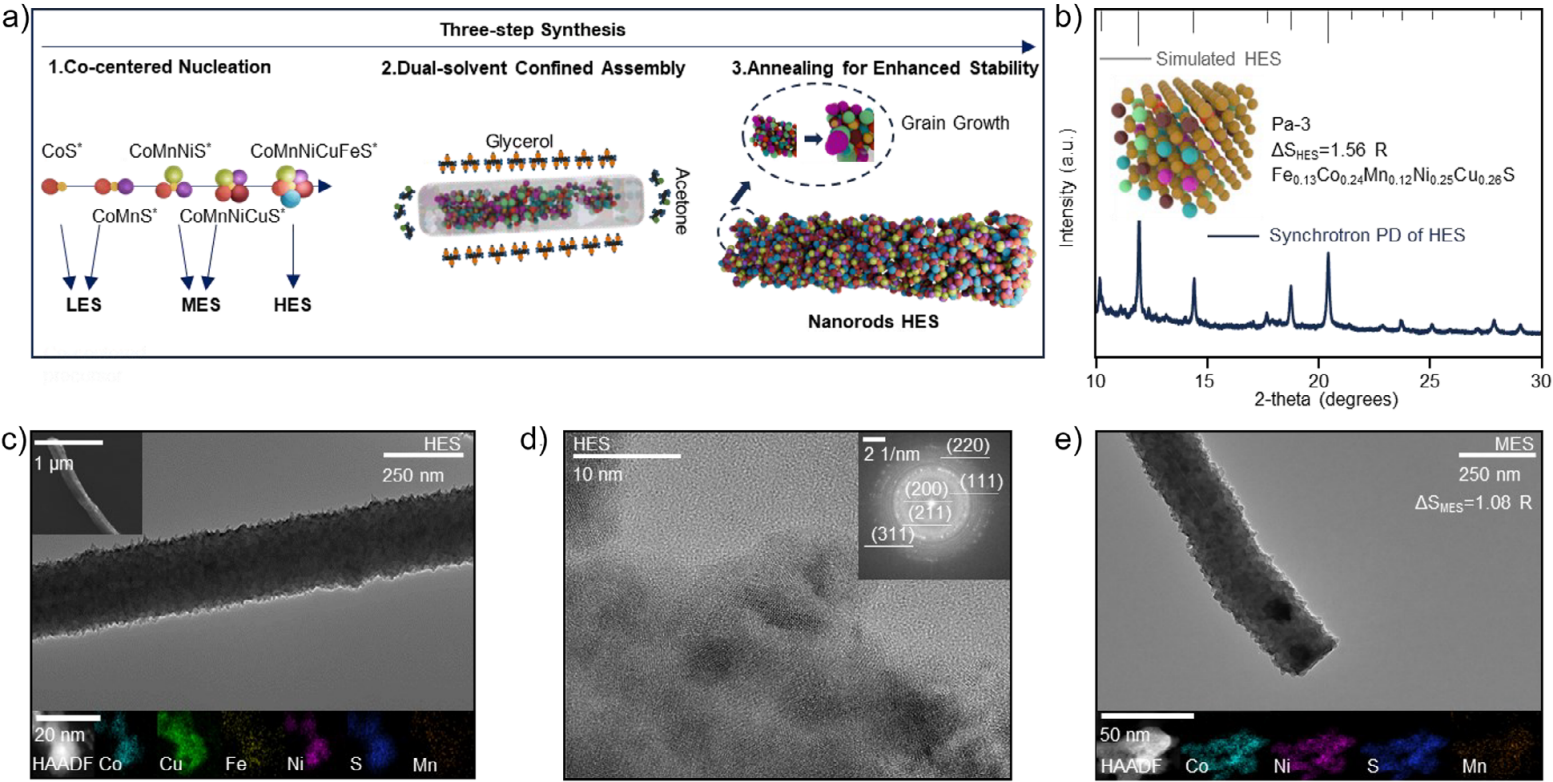
a) Schematic of the three‐step synthesis: Co‐centered nucleation, dual‐solvent confined assembly, and annealing. b) Operando synchrotron PD, simulated XRD patterns with inset of crystal model of HES nanorods. c) SEM and TEM images of HES nanorod and HAADF‐STEM mapping images of HES particles after ultrasonication. d) HRTEM and SAED images highlighting nanocrystalline features of HES particles after ultrasonication. e) TEM images of MES nanorod and HAADF‐STEM mapping images of MES particles after ultrasonication.

We employ a three‐step synthesis strategy to produce low‐, medium‐, and high‐entropy catalysts (Figures 1a and S1a–c). The first step is Co‐centered nucleation. Specifically, Co(acac)_2_ reacts with thioacetamide to form CoS^*^ precursor in acetone as the initial Co‐centered nuclei. Other components (Mn, Ni, Cu, Fe) are then sequentially introduced to produce precursors with increasing entropy levels, categorized by their configurational entropy as low‐entropy sulfide (LES, Co, S), medium‐entropy sulfide (MES, Co, Mn, Ni, S), and high‐entropy sulfide (HES, Co, Mn, Ni, Fe, Cu, S). The Co–Mn–Ni–Fe–Cu combination was selected due to the similar atomic radii of its constituent elements with that of zinc. This compatibility facilitates the formation of a homogeneous solid solution and ensures interfacial stability with ZnS. The configurational entropy (Δ*S*_conf_) is defined as low entropy (Δ*S*_conf_ < 1 *R*, where *R* is the universal gas constant), medium entropy (1 *R* < Δ*S*_conf_ < 1.5 *R*), and high entropy (Δ*S*_conf_ >1.5 *R*).^[^ ^8^ ^]^ Notably, in contrast to conventional Cu‐based cation exchange method, which frequently results in inhomogeneous intermediates, the initial CoS* nuclei in our strategy serve as catalytic seeds that trigger the codeposition of other metals, facilitating the uniform incorporation of all elements into a homogeneous high‐entropy solid solution.^[^ ^11^, ^12^ ^]^ In contrast, when Mn or Ni are used as initial nuclei, the formation of single‐phase compounds is observed (Figures S2a,b and S3).^[^ ^11^ ^]^ Moreover, CoS^*^ nuclei critically maintain the *Pa‐3* symmetry in the solid solution structure (Figure 1b). As shown in the XRD results (Figure S4), the absence of Co disrupts this *Pa‐3* structure. The second step involves a dual‐solvent assembly process. The as‐prepared low‐ to high‐entropy precursors assemble into nanorods within 0.5 h in a binary solvent system, achieving optimal morphology at 6 h. Nevertheless, extending the reaction to 10 h leads to over‐assembly (Figures S5a–c). When a single solvent is used, ethanol yields nanospheres, whereas glycerol results in the formation of nanosheets (Figures S6a,b).^[^ ^13^ ^]^ The final step of annealing promotes grain growth and enhances the mechanical stability of the catalysts. The morphology of the HES catalyst is characterized by scanning electron microscopy (SEM) and transmission electron microscopy (TEM). As shown in Figure 1c, the HES nanorods exhibit a diameter of 240 nm and a length of 4–5 µm, with homogeneously distributed metal elements.

To investigate the crystal features of the as‐prepared LES, MES, and HES catalysts, high‐resolution TEM (HRTEM) is employed. For the HES, the formation of nanocrystalline grains indicates the development of short‐range ordered structures, which is a typical feature of high‐entropy solid solutions (Figures S7a,b and S8).^[^ ^8^, ^14^ ^]^ The synchrotron PD pattern of HES catalysts closely aligns with the simulated XRD pattern, confirming their *Pa‐3* symmetry structure.^[^ ^15^ ^]^ Furthermore, ICPMS analysis (Table S1) reveals the chemical formula of the HES catalyst is Fe_0.13_Co_0.24_Mn_0.12_Ni_0.25_Cu_0.26_S, with a high configurational entropy of Δ*S*_conf_ = 1.56 *R* (12.96 J mol^−1^ K^−1^),^[^ ^8^ ^]^ implying its thermodynamic and electrochemical stability.^[^ ^8^ ^]^ The high‐angle annular dark field scanning transmission electron microscopy (HAADF‐STEM) results further reveal the uniform distribution of elements (Figure 1c). Among these, relatively weak Fe and Mn signals indicate higher oxidation states compared to Co, Cu, and Ni, which is consistent with the findings from the ICPMS analysis. Selected area electron diffraction (SAED) analysis shows distinct (111), (200), (211), (220), and (311) lattice planes of the HES catalysts, consistent with the synchrotron PD results (Figure 1d).

This three‐step synthesis method produces nanorod catalysts with tunable entropy levels—low‐, medium‐ (Figure 1e), and high‐entropy configurations. Among these, the HES catalyst exhibits uniform metal distribution, crystallographic integrity, and high configurational entropy. The HES is set as the experimental group, with MES as the primary control, to study the impact of catalyst's entropy on sulfur utilization and cycling stability of the fast‐charging AZSBs.

### Catalyzing RDS to Improve Sulfur Utilization

**Figure 2:**
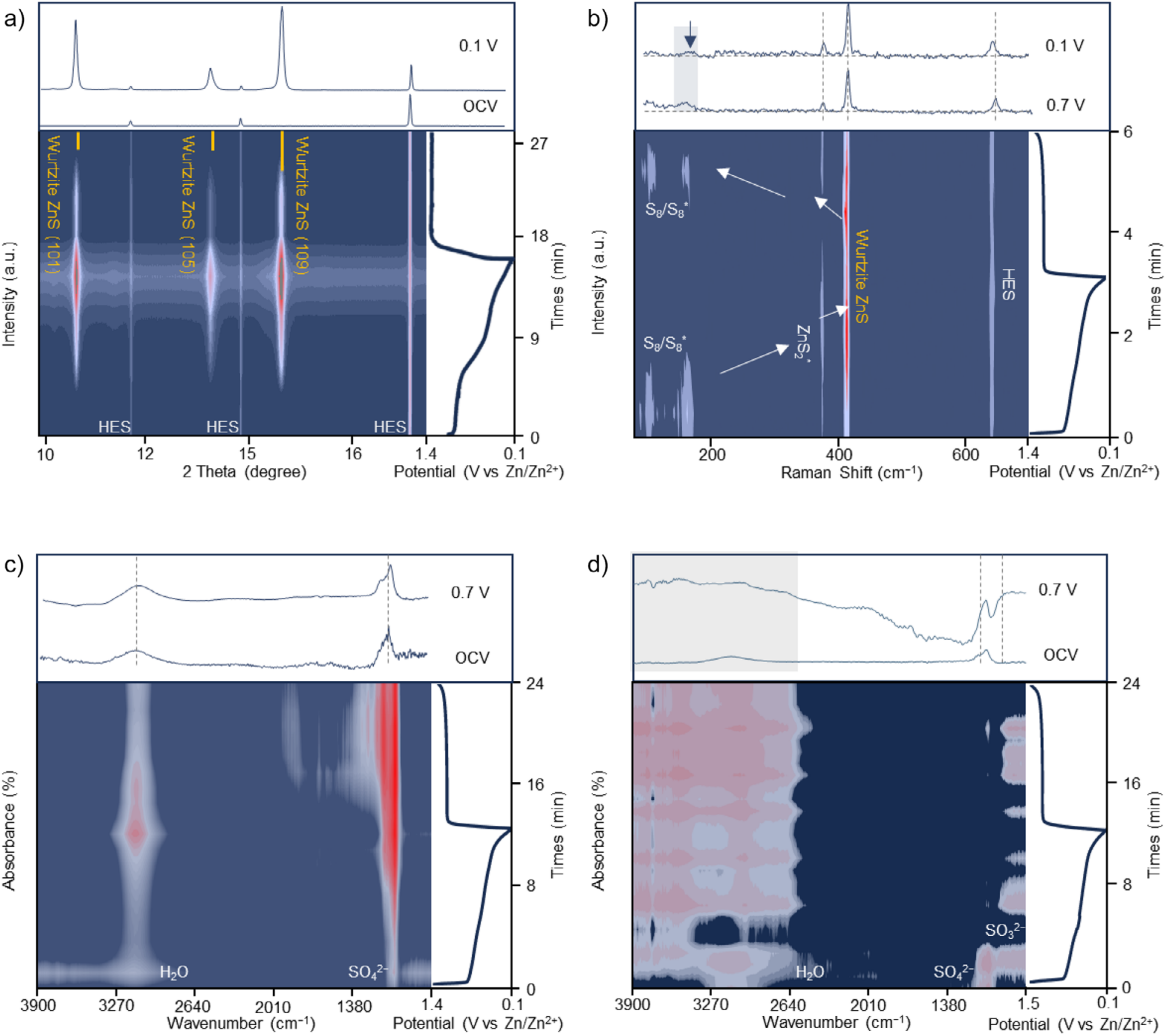
a) Operando synchrotron PD of the S@HES||Zn battery during cycling, showing the formation of wurtzite ZnS. b) Operando Raman spectra of the S@HES||Zn battery, revealing the reversible redox cycling between ZnS_2_^*^–ZnS. c) Operando synchrotron IRM data of the S@HES||Zn battery, presenting vibrational modes of SO_4_^2−^ (ν_1_ ∼ 1100 cm^−1^) and H_2_O (ν_1_ ∼ 3200 cm^−1^, ν_2_ ∼ 3600 cm^−1^). d) Operando synchrotron IRM data of the S@MES||Zn battery, presenting vibrational modes of H_2_O (ν_1_ ∼ 3200 cm^−1^, ν_3_ ∼ 3600 cm^−1^) and SO_3_^2−^ (ν_1_ ∼ 970 cm^−1^).

To identify and catalyze the RDS in the solid–solid Zn–S redox process, we implement the operando synchrotron PD, operando synchrotron IRM, and operando Raman characterizations by incorporating the MES and HES catalysts into S@MES||Zn and S@HES||Zn batteries. As shown in Figure 2a, distinct wurtzite ZnS peaks corresponding to the (101), (105), and (109) planes emerge during discharge in the S@HES battery and vanish upon charging. In contrast, sphalerite ZnS is the typical discharge product in the reported AZSBs.^[^ ^13^ ^]^ This wurtzite ZnS phase, as reported,^[^ ^16^ ^]^ can reduce the charging polarization with accelerated oxidation reaction within the Zn–S redox process to improve the sulfur utilization compared to sphalerite ZnS.^[^ ^17^ ^]^

Operando Raman spectroscopy provides insights into the different sulfur utilization between HES catalysts and MES catalysts. At the open‐circuit voltage of the S@HES||Zn battery, both S_8_ and S_8_^*^ species are detected. During early discharge, the peak at ∼380 cm^−1^ suggests the formation of ZnS_2_^*^ (Figure 2b).^[^ ^18^, ^19^, ^20^, ^21^ ^]^ Subsequently, the appearance of the peak at ∼410 cm^−1^ indicates the formation of wurtzite ZnS.^[^ ^22^ ^]^ Additionally, a persistent weak peak at ∼630 cm^−1^ is attributed to the HES catalysts. During charging, the redox reaction exhibits high reversibility with rapid conversion from wurtzite ZnS into ZnS_2_^*^ and ultimately into S_8_. Consequently, the complete reaction pathway catalyzed by HES can be summarized as: S_8_/S_8_^*^ ↔ ZnS_2_^*^ ↔ ZnS. For comparison, operando Raman tests of the S@MES||Zn battery reveal that although MES catalysts facilitate the conversion of ZnS_2_^*^, weak peaks in the 200–300 cm^−1^ range suggest the presence of irreversible intermediates, such as S_5_^2−^ and S_3_^2−^, during the discharge/charge cycle (Figure S9). The by‐product formation in S@MES||Zn batteries reduce sulfur utilization, leading to low coulombic efficiency. In addition, other weak peaks at 600–630 cm^−1^ may correspond to the potential leaching effects of the MES catalysts, indicating lower stability compared to the HES catalysts. Furthermore, operando synchrotron IRM spectra (Figures 2c,d) reveal that only SO_4_^2−^ (ν_1_) and H_2_O (ν_1_, ν_3_) bands are detected in the S@HES|||Zn battery, indicating that HES catalysts efficiently enhance sulfur utilization. Notably, irreversible sulfur intermediates and sulfur disproportionation products, commonly reported in many AZSBs, are absent in the S@HES||Zn battery. In contrast, SO_3_^2−^ species (∼970 cm^−1^) are observed in the S@MES||Zn battery, which is identified as a by‐product due to the sulfur disproportionation reaction.^[^ ^23^ ^]^ These findings highlight that HES catalysts optimize the reaction pathway of the solid–solid Zn–S redox process, ensuring efficient and rapid sulfur conversion. In contrast, MES catalysts lead to formation of sulfur‐containing by‐products, resulting in incomplete conversion of S_8_ to wurtzite ZnS during discharging and decreased sulfur utilization.

These results reveal that HES catalysts exhibit high sulfur utilization of AZSBs by efficiently optimizing the reaction pathway of the solid–solid Zn–S redox process. In contrast, MES catalysts exhibit low sulfur utilization due to by‐product formation. This distinction in sulfur utilization can be attributed to the high‐entropy cocktail effect. Specifically, the five‐component system of HES catalysts enables a diverse and synergistic distribution of active sites, significantly accelerating the solid–solid Zn–S redox process compared to the three‐component system of MES catalysts.^[^ ^14^ ^]^

### Relationship Between Entropy and Cycling Stability

As implied in Raman results, HES catalysts exhibit higher cycling stability compared to MES catalysts because of the leaching effects in the MES.^[^ ^8^ ^]^ To further explore the relationship between catalysts’ entropy and cycling stability, NEXAFS, ICP‐MS, and electronmicroscopic characterizations were conducted to examine the structural changes of the catalysts in S@HES and S@MES cathodes.

**Figure 3:**
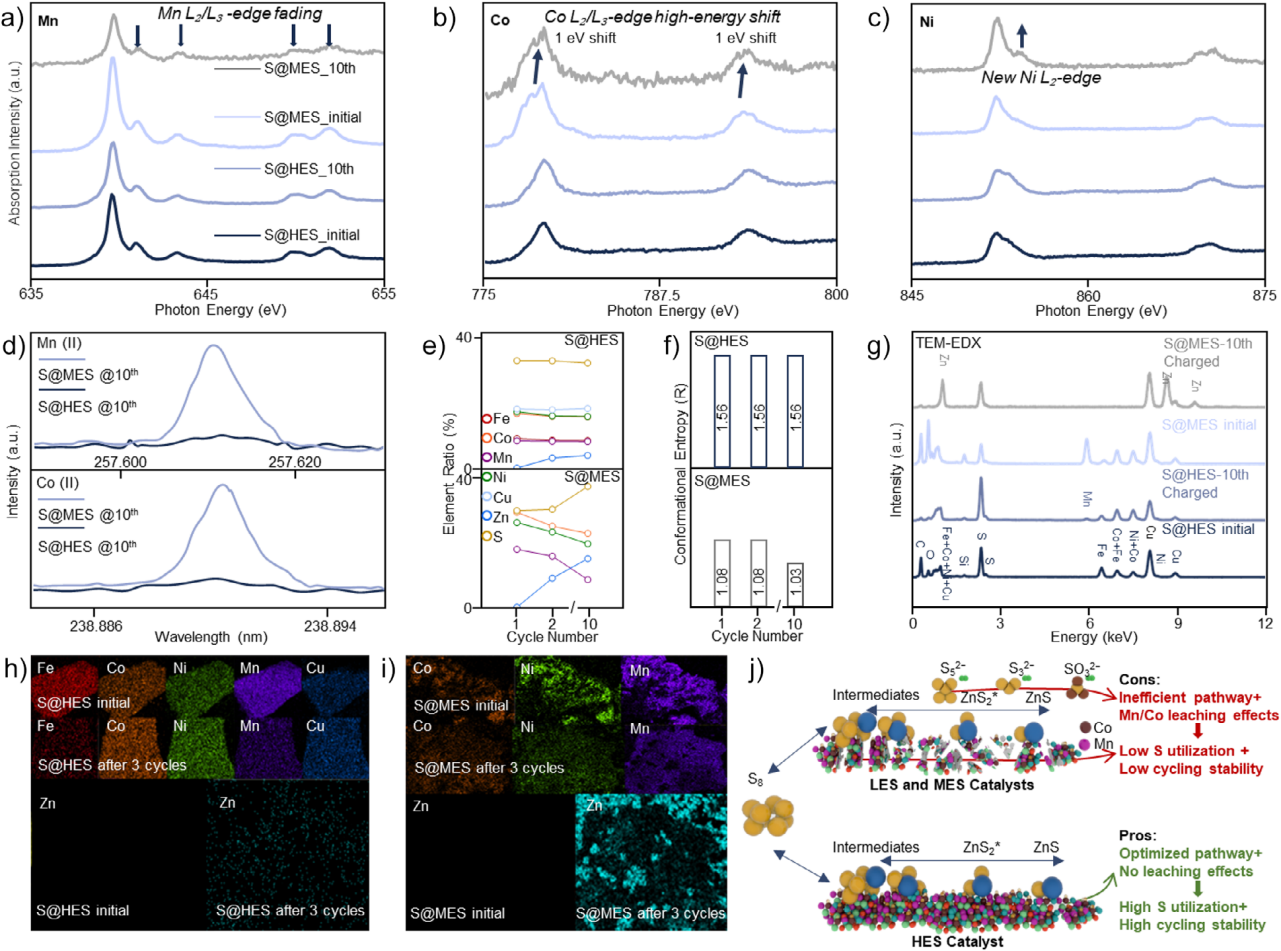
NEXAFS spectra of a) Mn, b) Co, and c) Ni for S@HES||Zn and S@MES||Zn batteries, measured before and after 10 cycles. d) Liquid ICPMS analysis of the electrolyte from S@HES||Zn and S@MES||Zn batteries after 10 cycles to evaluate Mn(II) and Co(II) dissolution. e) and f) Elemental content and structural configuration changes before and after cycling for S@HES||Zn and S@MES||Zn batteries. g)–i) TEM‐EDX and mapping analysis of S@HES||Zn and S@MES||Zn batteries before and after 3 cycles, showing residual unoxidized ZnS on the S@MES electrode surface after charging. j) Schematic illustration of the impact of catalysts’ entropy on sulfur utilization and cycling stability.

The NEXAFS results demonstrate that the chemical states of Mn, Co, Ni, and Fe in the S@HES cathodes are highly stable throughout cycling (Figures 3a–c and S10). In contrast, the chemical states of Mn, Co, and Ni in the S@MES cathodes undergo substantial change during cycling. Specifically, the intensities of Mn L_2_ edge at 650 eV and L_3_ edge at 642 eV exhibit a notable decrease, dropping to 41% of their original intensity, which indicates a significant reduction in the chemical state of Mn after 10 cycles. The Co L_2_ edge at 793 eV and Co L_3_ edge at 778 eV edges slightly shift toward higher energy, appearing at new positions of Co L_2_ edge (ca. 794 eV) and Co L_3_ edge (ca. 779 eV) edges, whereas a new Ni L_2_ edge at 870 eV emerge after 10 cycles, indicating an increase of the chemical states for both Co and Ni. These observations suggest potential metal leaching in the MES catalysts, wherein the three‐component system (Co, Mn, Ni) compensates for the dissolution of one metal species through oxidation state adjustments among the remaining elements to preserve overall electroneutrality.

To gain deep insight into the leaching effects, liquid ICPMS analysis of the electrolytes after 10 cycles was further conducted (Figure 3d). The study reveals Mn (II) and Co (II) leaching into the electrolyte in the S@MES||Zn battery, whereas these signals are not observed in the electrolyte of the cycled S@HES||Zn battery. In addition, ICPMS results illustrate the direct element changes in HES and MES catalysts after 10 cycles (Figures 3e,f). The configurational entropy of HES catalysts remains stable at 1.56 *R*, whereas the configurational entropy of MES catalysts significantly decreases from 1.08 *R* to 1.03 *R* (Table S2). TEM‐EDX and corresponding mappings (Figures 3g–i) reveal the presence of “dead ZnS”^[^ ^13^ ^]^ in the S@MES cathodes at the charged status, referring to residual ZnS that cannot be reconverted to S during charging, which reduces the configurational entropy. Therefore, this decrease in the configurational entropy is primarily due to Mn/Co leaching, with a secondary contribution from the accumulation of ZnS residues.

To investigate the suppression capability of water splitting from low‐entropy to high‐entropy catalysts, CV and in situ GCMS analyses are conducted in batteries with varying entropy levels: S@LES||Zn, S@MES||Zn, and S@HES||Zn systems. The S@HES||Zn battery exhibits the highest electrochemical stability within the voltage range of 0.05–1.40 V (Figure S11), with no signals indicating leaching effects or water splitting. In contrast, distinct peaks emerged during the initial reduction reaction in the S@LES||Zn and S@MES||Zn batteries, indicating the Mn/Co leaching effects corresponding to the liquid ICPMS results. Additionally, in situ GCMS results confirm that, apart from the residual oxygen initially present in the electrolytic cell, no detectable oxygen accumulated in the S@HES||Zn batteries over five cycles at a current density of 20 A cm^−2^(Figure S12).

Overall, the above analyses confirm a correlation between high entropy and cycling stability, driven by the high‐entropy effect.^[^ ^14^ ^]^ This means high‐entropy materials can stabilize high‐entropy solid solution structures and suppress leaching effects and water splitting. Figure 3j summarizes a comprehensive understanding of the impact of catalysts’ entropy on sulfur utilization and cycling stability. HES catalysts optimize the Zn–S redox pathway and maintain structural stability, achieving high sulfur utilization and cycling stability. In contrast, LES and MES catalysts exhibit inefficient pathways, leading to by‐products and Mn/Co leaching, thus reducing sulfur utilization and cycling stability.

### Electrochemical Performances of Fast‐Charging AZSBs

**Figure 4:**
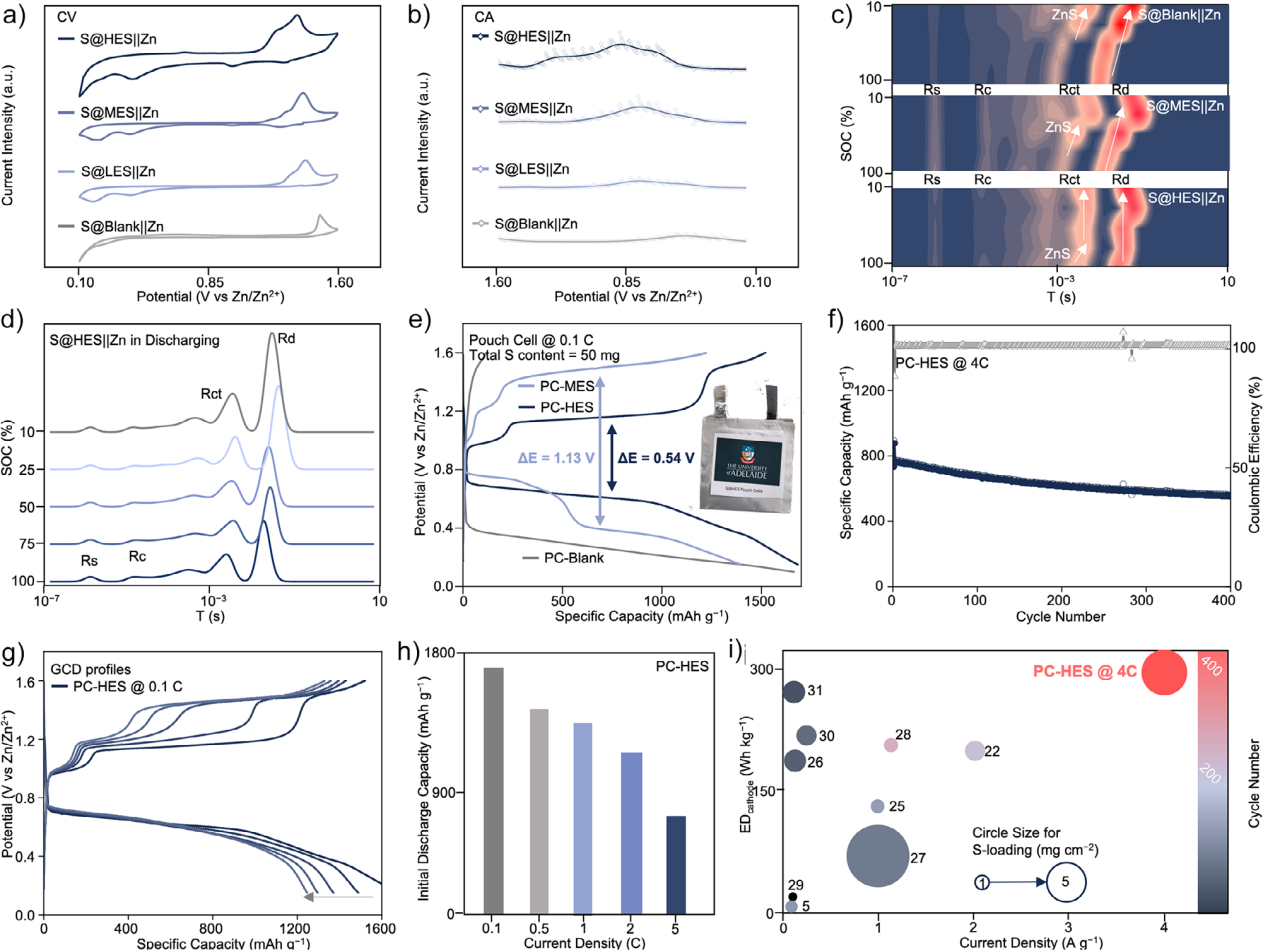
a) CV and b) steady state CA profiles for S@blank||Zn, S@LES||Zn, S@MES||Zn, and S@HES||Zn batteries. c) In situ DRT images of S@blank||Zn, S@MES||Zn, and S@HES||Zn batteries. d) In situ DRT curves of S@HES||Zn batteries. e) GCD curves of PC‐HES, PC‐MES, and PC‐Blank at 0.1 C with the inset of the photograph of fabricated pouch cell. f) Fast‐charging cycling tests of PC‐HES at 4 C. g) GCD profiles of PC‐HES in the 1st, 10th, 20th, 30th, and 50th cycle at 0.1 C, respectively. h) Initial discharge capacity distribution of the PC‐HES at different rates. i) Performance metrics between recent pouch‐cell AZSBs, showing superior lifespan, cathode energy density, current density, and sulfur loading.^[^ ^5^, ^22^, ^25^, ^26^, ^27^, ^28^, ^29^, ^30^, ^31^ ^]^

To demonstrate the practical applications of the mechanisms, we evaluate the electrochemical performances of AZSBs with LES, MES, HES catalysts and without catalysts. Electrochemical CV profiles demonstrate that as the entropy increases, the reduction peaks shift to higher voltages, and the oxidation peaks shift to lower voltages, reducing the overall polarization (Figure 4a).^[^ ^24^ ^]^ In addition, derived from chronoamperometry (CA) results shown in Figure S13, it confirms that high entropy catalysts exhibit early onset potentials for the discharge reaction (Figure 4b). These findings underscore the rapid conversion kinetics of the solid–solid Zn–S redox process.

The in situ distribution of relaxation times (in situ DRT) results further reveal the impedance evolution of S@blank||Zn, S@MES||Zn, and S@HES||Zn batteries under various states of charge (SoCs) (Figures 4c,d, S14, and S15). At the early discharge stage (0.70 V vs. Zn^2+^/Zn), the S@HES||Zn battery exhibits a slight increase in charge transfer resistance (*R*_ct_), demonstrating the rapid ZnS conversion process. In contrast, a steep rise in *R*_ct_ is observed during the middle and late discharge stages in the S@MES||Zn and S@blank||Zn batteries, indicating that the ZnS conversion in these two systems occurs at a lower reduction potential, resulting in higher battery polarization compared with the S@HES||Zn batteries. In addition, the similar changes in Warburg diffusion resistance (*R*_d_) further emphasize the superior Zn–S redox kinetics of the S@HES||Zn battery.

To evaluate the practical electrochemical performance, pouch cells with various catalysts were assembled and designated as PC‐blank, PC‐MES, and PC‐HES, as shown in Figure S16. Galvanostatic charge–discharge (GCD, Figure 4e) measurements reveal that the PC‐HES battery exhibits a low polarization voltage of Δ*E* = 0.54 V at 0.1 C. Notably, the PC‐HES system maintained a stable discharge plateau at 0.61 V, achieving a high cathode energy density of 313 Wh kg^−1^ (based on the mass of sulfur, catalysts, carbon, and binder). During the charging process, PC‐HES displays two distinct plateaus at 0.95 and 1.17 V, corresponding to the activation of the thick wurtzite ZnS layer and its subsequent oxidation to sulfur as confirmed by previous CV analyses. The coin‐type S@HES||Zn battery (Figures S17 and S18) demonstrated a higher discharge plateau at 0.75 V and a single charge plateau at 1.17 V, resulting from the thinner insulating layer of the sulfur species compared to that in pouch cells. In contrast, PC‐MES exhibits two separated discharge plateaus at 0.70 and 0.35 V, indicating increased polarization associated with an alternative reaction pathway. This facilitates the formation of undesirable polysulfides by‐products such as S_5_^2−^ and S_3_^2−^ species, compromising battery performance. This observation aligns well with the results from the in situ DRT, CV, and CA analyses.

The fast‐charging capability of PC‐HES was evaluated at a high current rate of 4 C (Figure 4f). The cell delivered an initial reversible capacity of 770 mAh g^−1^ and retained 577 mAh g^−1^ after 400 cycles, corresponding to a capacity retention of 75%. This performance reflects an exceptionally low‐capacity decay rate of 0.06% per cycle, as further confirmed by the GCD profiles in Figure S19. In addition to fast‐charging performance, the low‐rate performance and corrosion resistance of PC‐HES were systematically investigated. As shown in Figure 4g, PC‐HES exhibits excellent cycling stability at 0.1 C for 50 cycles, as indicated by the consistent discharge plateau at 0.61 V, demonstrating sustained catalytic activity of the HES. More importantly, as shown in Figures S20a,b, corrosion tests highlight the superior stability of the PC‐HES, which maintained electrode integrity owing to its nonleaching characteristics. In contrast, conventional iodine‐based AZSBs suffer from pronounced zinc corrosion. Figure S21a presents a comparative analysis of the rate performance for PC‐MES and PC‐HES, with corresponding GCD profiles shown in Figure S21b. The PC‐HES system demonstrates stable capacity retention across a range of rates from 0.1 to 2 C, exhibiting only moderate capacity fading at the high rate of 5 C, as shown in Figure 4h. In contrast, PC‐MES displays continuous capacity degradation over the 0.1–2 C range, which is attributed to the gradual leaching of Mo/Co catalytic components during cycling.

Compared with recently reported pouch‐type AZSBs employing liquid redox mediators (Figure 4i and Table S3), the PC‐HES system exhibits superior performance across several key metrics. Notably, it delivers a high cathode energy density of 313 Wh kg^−1^ with a stable 400 cycles at 4 C under a practical sulfur loading of 50 mg per cell. Moreover, the system exhibits an exceptionally low capacity decay of only 0.5 mAh g^−1^ per cycle, highlighting its strong potential for high‐performance and durable energy storage applications.

## Conclusion

In summary, we synthesized the HES catalyst through a three‐step process and achieved high sulfur utilization and cycling stability because of the optimized reaction pathway and suppressed leaching effects and water splitting side reactions. Operando synchrotron techniques confirm that HES catalysts efficiently catalyze the RDS conversion from ZnS_2_^*^ to wurtzite ZnS without forming by‐products, including S_5_^2−^, S_3_^2−^, and SO_3_^2−^. Furthermore, NEXAFS and ICPMS analyses reveal a correlation between high entropy and cycling stability. Consequently, the assembled pouch cells with HES catalysts sustain over 400 cycles at a fast‐charging current density of 4 C with only 0.06% capacity loss per cycle. This work establishes an entropy‐driven catalytic strategy to address sluggish solid–solid redox kinetics in AZSBs and provides a fast‐charging solution for next‐generation aqueous metal–sulfur batteries.

## Supporting Information

The authors have cited additional references within Supporting Information.^[^ ^17^ ^]^

## Conflict of Interests

The authors declare no conflict of interest.

## Supporting information



Supporting Information

## Data Availability

The data that support the findings of this study are available from the corresponding author upon reasonable request.
