# Supplementary material for: High‐Entropy Sulfides Catalyze Rate‐Determining Redox in Fast‐Charging Aqueous Zinc–Sulfur Batteries
Source: Angew Chem Int Ed Engl. 2025 May 13;64(28):e202503472. doi: 10.1002/anie.202503472 (PMC12232885; doi:10.1002/anie.202503472)
Supplement: Supplementary file 1 — Supporting Information [file ANIE-64-e202503472-s001.docx]

SUPPORT INFORMATION

**High-Entropy Sulfides Catalyze Rate-Determining Redox in Fast-charging Aqueous Zinc-Sulfur Batteries**

Jiahao Liu,^a^ Han Wu,^a^ Chao Ye,^a^* and Shi-Zhang Qiao^a^*

^a^ School of Chemical Engineering, The University of Adelaide, Adelaide, SA 5005, Australia

*Email: [chao.ye@adelaide.edu.au](mailto:chao.ye@adelaide.edu.au); [s.qiao@adelaide.edu.au](file:///C:\Users\Administrator\Desktop\s.qiao@adelaide.edu.au)

**Experimental Procedures**

**Chemicals:** Zinc sulfate hexahydrate (ZnSO_4_·6H_2_O, ≥ 99.0 %), Cobalt acetylacetonate (Co(C_5_H_7_O_2_)_3_ ≥ 99.0 %), Copper acetylacetonate (Cu(C_5_H_7_O_2_)_2_, ≥ 99.9 %), Iron acetylacetonate (Fe(C_5_H_7_O_2_)_3_ ≥ 99.9 %), Nickel acetylacetonate (Ni(C_5_H_7_O_2_)_2_, ≥ 95.0 %), Manganese acetylacetonate (Mn(C_5_H_7_O_2_)_2_, ≥ 95.0 %), thioacetamide (TAA, ≥ 99.0 %), sulfur (≥ 99.98 %), selenium (≥ 99.99 %), polytetrafluoroethylene preparation (PTFE, 60% wt dispersion in H_2_O), glycerol and acetone (CH_3_COCH_3_, ≥ 99.5 %) were purchased from Sigma-Aldrich. Ethanol (undenatured 100%) was purchased from ChemSupply, Australia. Ketjen Black (KB, ECP-600JD) was purchased from Lion Corporation. Carbon black, Super P® conductive (≥ 99.0 %) was purchased from Alfa Aesar. Zn foil was purchased from Shenzhen Kejing Star Technology. Carbon cloth was purchased from Fuel Cell Battery.

**Synthesis of Low- to High-entropy Sulfides:** In a typical autocatalytic synthesis, 71.3 mg of Co(C_5_H_7_O_2_)_3_ was added to 10 mL of acetone and stirred using a magnetic stirrer at 500 rpm for 5 minutes. Subsequently, 52.4 mg mol of Cu(C_5_H_7_O_2_)_3_, 70.6 mg Fe(C_5_H_7_O_2_)_3_, 51.4 mg Ni(C_5_H_7_O_2_)_2_, and 50.6 mg Mn(C_5_H_7_O_2_)_2_ were sequentially added with a syringe, followed by stirring for 30 minutes. Then, 75.13 mg of TAA was dissolved in 12 mL of acetone and added to the above solution, with stirring continued for another 30 minutes. Considering evaporation, acetone was replenished to a total volume of 20 mL, after which 20 mL of glycerol was immediately added, and stirring continued for an additional 30 minutes. The mixture was then transferred to a hydrothermal reaction vessel. Solvothermal treatment was carried out at 160°C in a constant temperature oven for 6 hours to obtain the optimal nanorod-shaped HES. The remaining LES and MES were synthesized using the same method.

**Characterization:** Transmission electron microscopy (TEM) and selected area electron diffraction (SAED) analyses were conducted on an FEI Tecnai G2 Spirit TEM at 120 kV. High-resolution TEM (HRTEM) was conducted on the FEI Titan Themis 80-200 instrument. A scanning electron microscope (SEM) and X-ray Energy Dispersive Spectroscopy (EDS) tests were implemented on an FEI Quanta 450 FEG device operating at 20 kV. X-ray powder Diffraction (XRD) data were collected on a Rigaku MiniFlex 600 instrument, driving at 40 kV, 15 mA. Simulated XRD patterns were generated using Co Kα radiation and simulated with CrystalDiffract software based on ICP-MS data. The Distribution of Relaxation Times (DRT) was calculated by in-situ electrochemical Impedance Spectroscopy (EIS) data performed on the Biologic EC-Lab electrochemical station. The metal element concentrations in the samples were determined using an Agilent 7900x inductively coupled plasma mass spectrometer (ICP-MS) attached to the New Wave NER213 laser ablation system. The electrolyte ICP-MS was conducted with Agilent 8900x QQQ-ICP-MS system. The test solution was diluted with a 2% nitric acid aqueous solution by volume. The in-situ gas chromatography-mass spectrometry (GC-MS) was performed on the Agilent GC at 20 A g^−1^ with Ar as the carrier gas.

**Operando Synchrotron Powder Diffraction (PD) Measurement:** The punched coins were sealed with transparent tape. Operando synchrotron PD data were collected at the powder diffraction beamline of the Australian Synchrotron (ANSTO) using two different wavelengths: 0.5903 Å for powder samples and 0.6888 Å for operando coin-type batteries, respectively. The operando PD for HES and MES AZSBs were performed for an entire cycle from OCV to 0.05 V and finally to 1.40 V.

**Operando Synchrotron Infrared Microspectroscopy (IRM) Measurement:** Operando synchrotron IRM was performed using the ATR mode of a Bruker VERTEX V70 spectrometer at the beamline of the ANSTO, covering the wavenumber range of 750-3900 cm^−1^. A CaF_2_ crystal and an open in-situ reaction cell were used, which was connected to a CHI 660E electrochemical workstation.

**Soft X-Ray Spectroscopy Measurement:** The near-edge X-ray absorption fine structure (NEXAFS) data of the Mn*_L-edge_* (*c.a.* 640 eV), Co*_L-edge_* (*c.a.* 780 eV), Ni*_L-edge_* (*c.a.* 850 eV), Fe*_L-edge_* (*c.a.* 710 eV) were collected by the soft X-Ray beamline at the Australian Synchrotron. The electrodes of charged and discharged states were removed from the disassembled batteries for the *ex-situ* test.

**Operando Raman Measurement:** The operando inVia^TM^ confocal Raman test was combined with the CHI760E electrochemical station. An operando test model from GaossUnion was utilized to detect the electrolyte near the cathode interface from *c.a.* 100 cm^−1^ to 800 cm^−1^.

**Electrochemical Measurements:** Using the standard method, the 2032 coin-type AZSB cells were prepared by mixing KB and sulfur at a 1:1 ratio, followed by baking in a vacuum-sealed glass tube in an oven at 160°C for 600 minutes. LES, MES, and HES catalysts were mixed with sulfur at a 1:20 ratio, ground evenly, and anchored on a stainless steel mesh using a 10 wt.% PTFE solution, with an average loading of 2-3 mg cm^−2^. Using the spray-coating method, KB and sulfur were mixed at a 1:1 ratio, with the catalyst accounting for 10% of the sulfur content. A suitable amount of CS_2_ was added and stirred evenly before being placed in a spray gun. The mixed solution was sprayed evenly onto carbon cloth with the spray gun while wearing a gas mask. The carbon cloth was folded with the active side inward and placed in a hydrothermal reaction vessel, followed by baking in an oven at 160°C for 10 h. The carbon cloth was then cut into appropriately sized electrode sheets, with an average loading of 2 mg cm^−2^ for thin coatings and 8 mg cm^−2^ for thick coatings. The typical pouch cell was constructed with a cathode, separator, anode, Ni tabs, and aluminum-plastic film. The cathode slurry was prepared by mixing 100 mg of sulfur-carbon composite powder, 10 mg of HES catalyst, 30 mg of conductive carbon, and 15 mg of PTFE in anhydrous ethanol, followed by grinding for 15 min. The resulting slurry was uniformly coated onto both sides of a Ti mesh using a roller press and then dried at 60°C for 4 h in an oven. The dried cathode sheet was further compressed with a hydraulic press to improve conductivity. A 0.03 mm thick high-purity Zn foil was used as the anode, while a Glassfiber-D membrane served as the separator. The cathode and anode sheets were welded with Ni tabs and vacuum-sealed in aluminum-plastic film at 190°C to fabricate a mechanically robust pouch cell. During the cycling and rate test, LAND CT2001 was performed from 1.40-0.05 V *vs.* Zn/Zn^2+^ at 0.1-5 A g^−1^ at 25 ^o^C, respectively. Cyclic voltammetry and chronoamperometry were performed using a CHI760 electrochemical workstation in a voltage range of 0.05-1.40 *vs.* Zn/Zn^2+^. The steady-state chronoamperometry (CA) was conducted at the Biologic Vmp-3e. The voltage range was set between 1.40-0.10V with a step size of 0.05V, and a step relaxation time of 300s was employed to allow the steady-state exchange current density to recover.

Supplementary Results


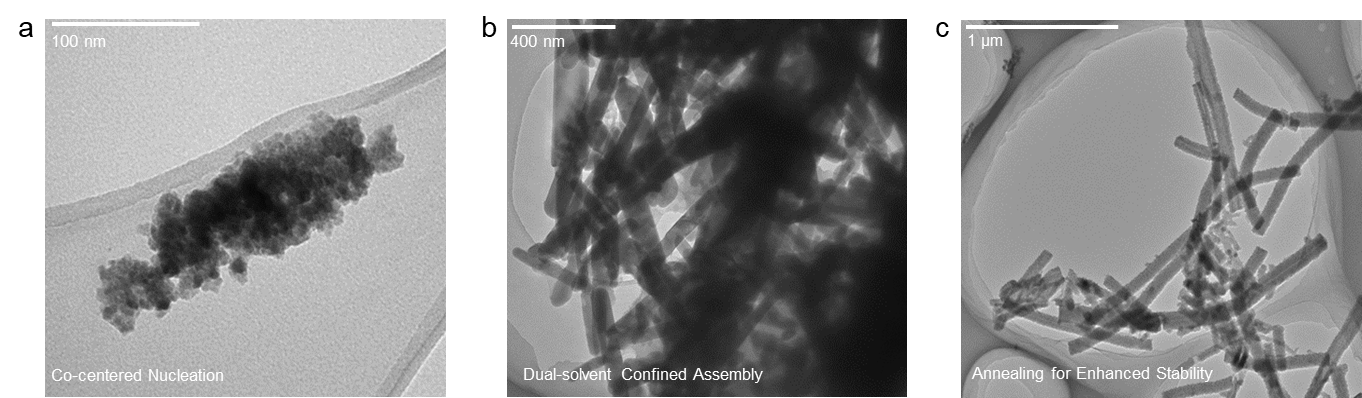


**Figure S1.** TEM images of the three-step synthesis: a) Co-centered nucleation, b) dual-solvent confined assembly, and c) annealing for enhanced stability.

**
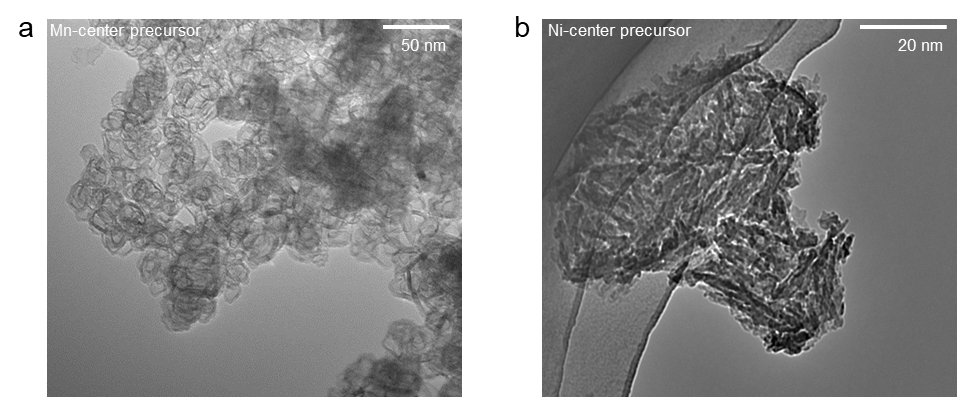
**

**Figure S2.** TEM images of a) Mn-centered precursors with cubic crystallinity and b) Ni-centered precursors with hexagonal crystallinity.

**
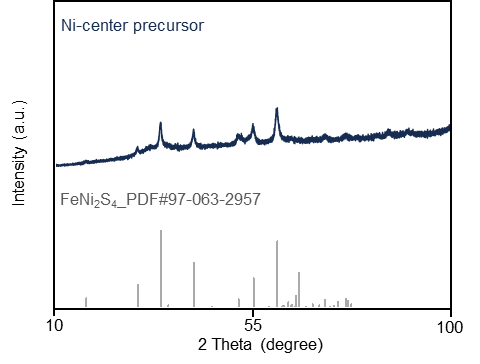
**

**Figure S3.** XRD results of HES from Ni-centered precursors showing similarity to FeNi_2_S_4_.

**
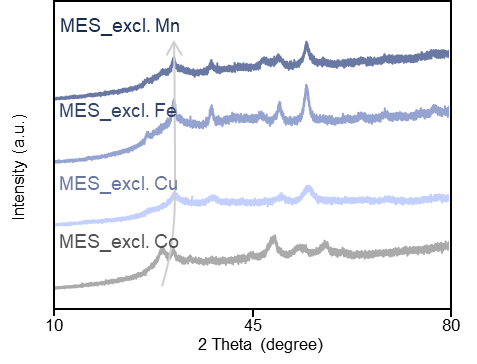
**

**Figure S4.** XRD results of MES-deficient components: Mn, Fe, and Cu deficiencies do not affect solid solution formation, whereas Co deficiency disrupts it.

**
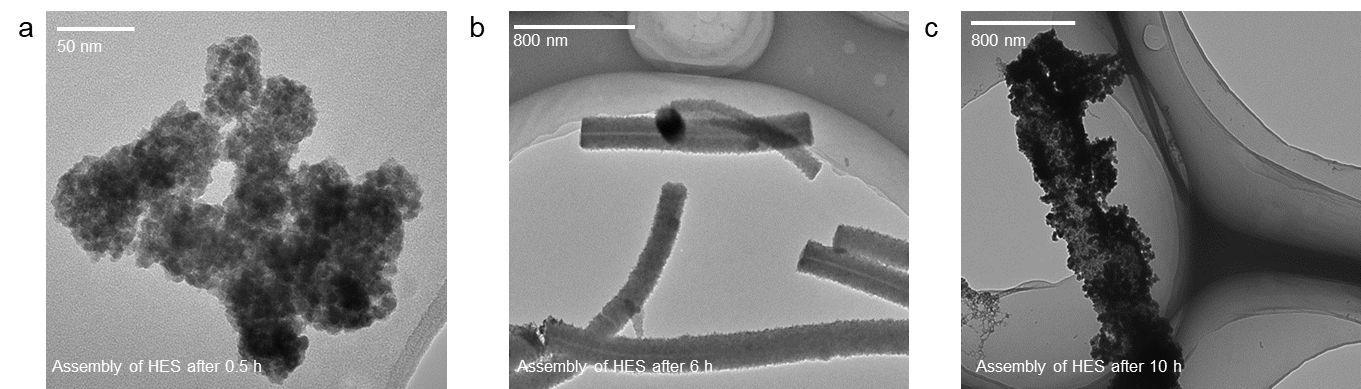
**

**Figure S5.** TEM images showing the solvothermal assembly of HES in acetone and glycerol mixed solvent: a) 0.5 h with initial nanorod formation, b) 6 h with optimal nanorod morphology, c) 10 h with over-assembly.

**
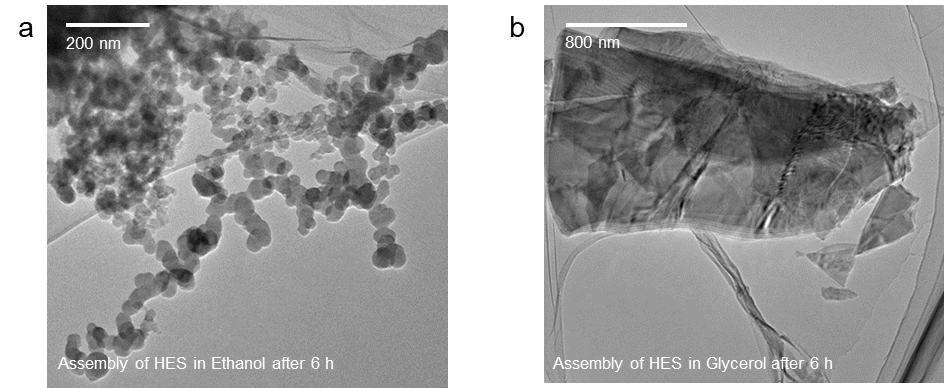
**

**Figure S6.** TEM images showing the solvothermal assembly of HES in single solvents: a) Nanospheres in ethanol and b) 2D nanosheets in glycerol.

**
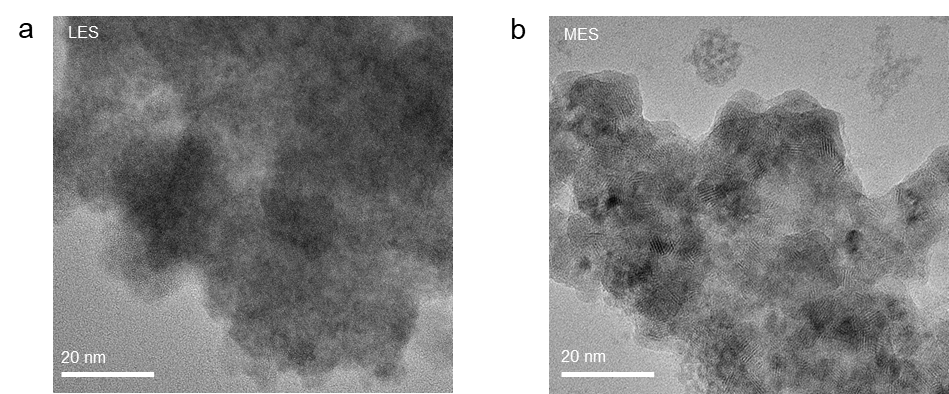
**

**Figure S7.** HRTEM images of catalysts of low- (a) and medium-entropy (b) after ultrasonication.

**
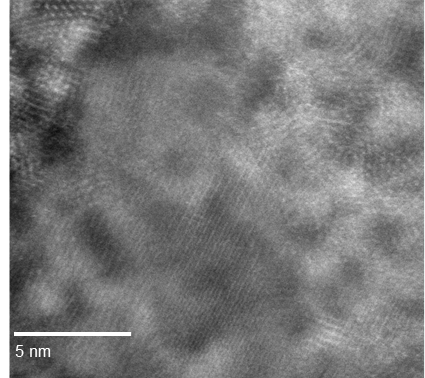
**

**Figure S8.** HRTEM image of HES catalyst after ultrasonication, highlighting typical high-entropy nanocrystalline regions.

**
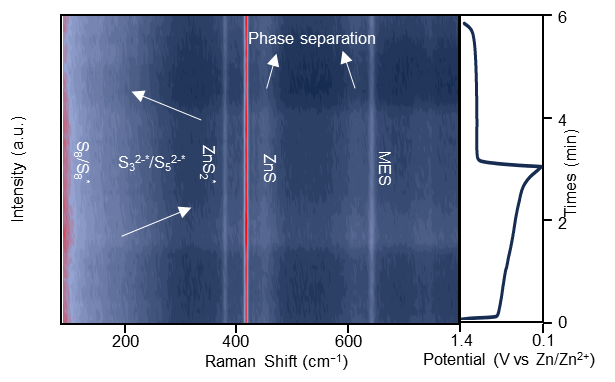
**

**Figure S9.** Operando Raman spectrum of the S@MES||Zn battery demonstrates similar catalytic capability but shows issues with phase separation and by-product formation.

**
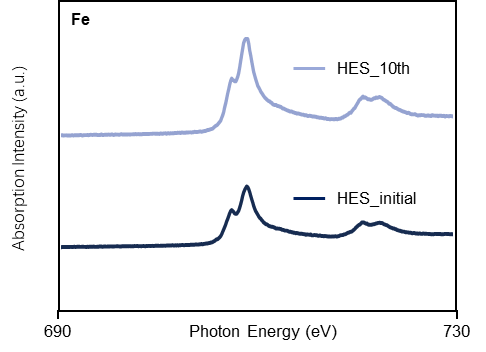
**

**Figure S10.** *Ex-situ* Fe NEXAFS spectra before and after 10 cycles for S@HES||Zn battery.

**
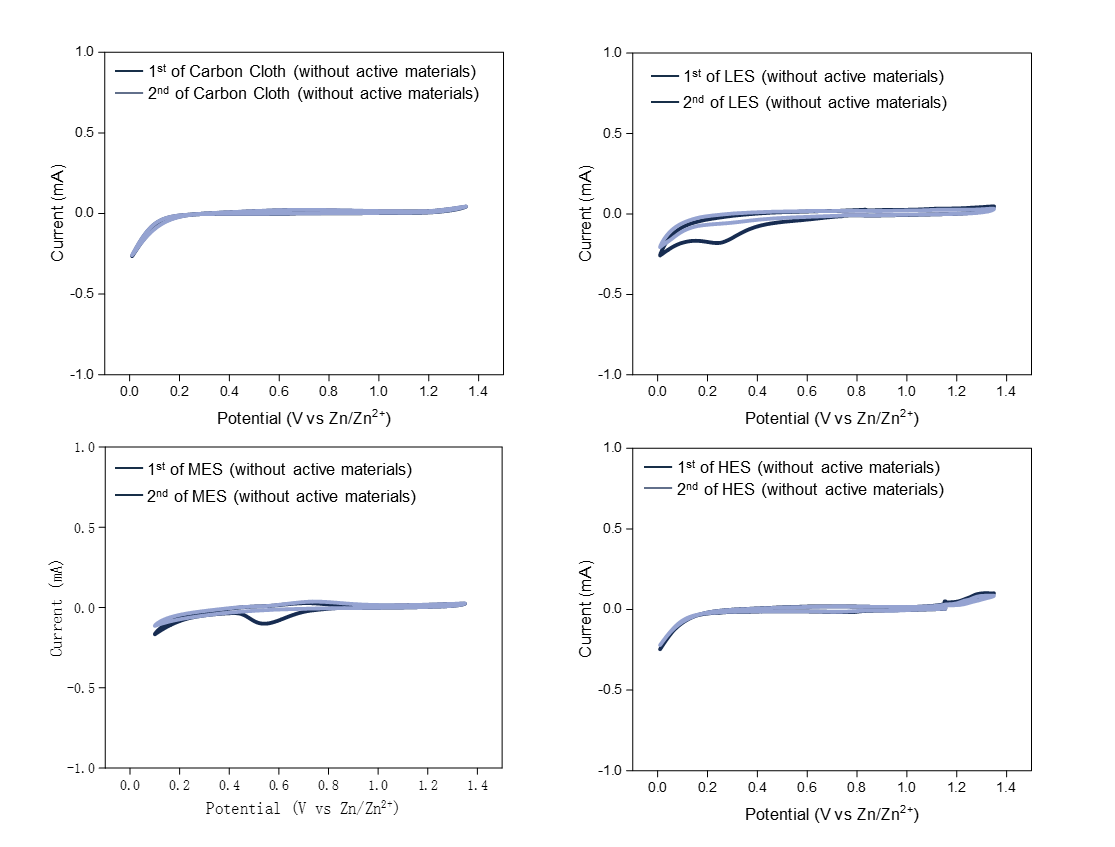
Figure S11.** CV stability tests of (a) blank carbon paper, (b) LES||Zn, (c) MES||Zn, and (d) HES||Zn batteries without sulfur as the active material.

**
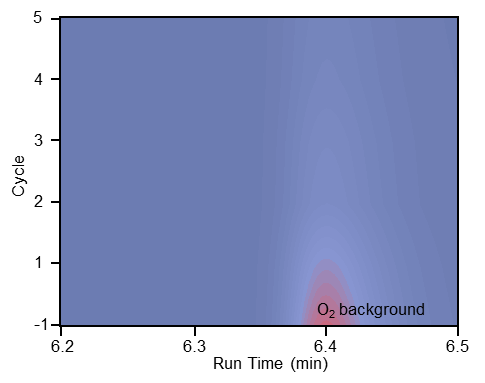
**

**Figure S12.** *In-situ* GC-MS result of S@HES||Zn batteries in 5 cycles at 20 A g^−1^, indicating no obivious OER. The initial O_2_ signal derives from the battery module.

**
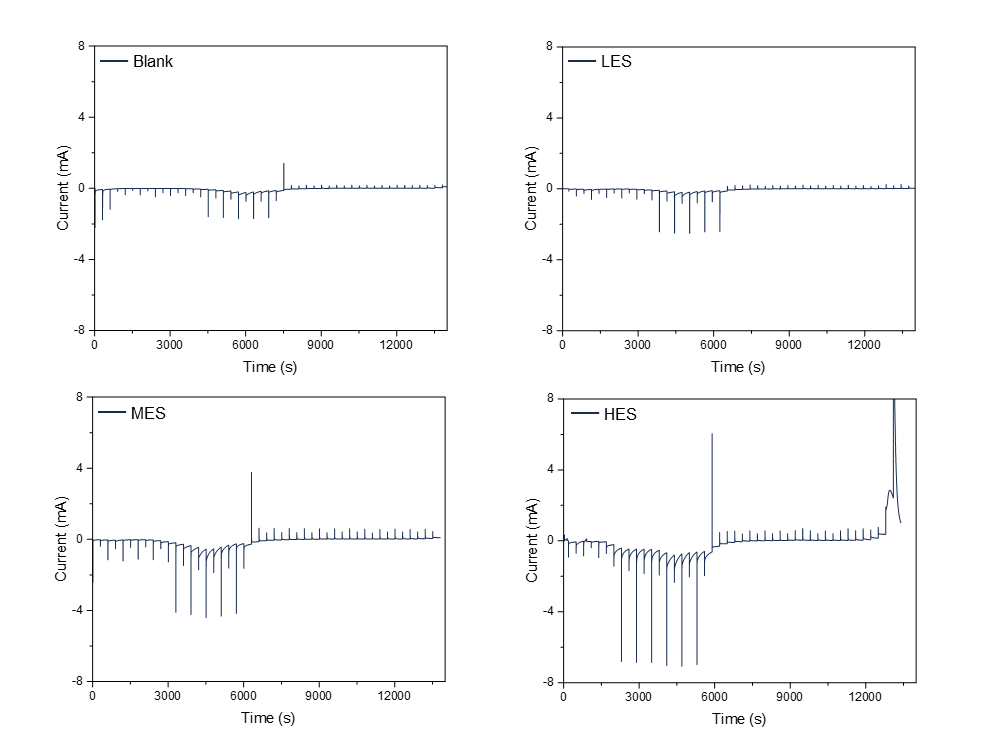
**

**Figure S13.** Steady-state CA profiles of blank sample, S@LES||Zn, S@MES||Zn, and S@HES||Zn batteries.

**
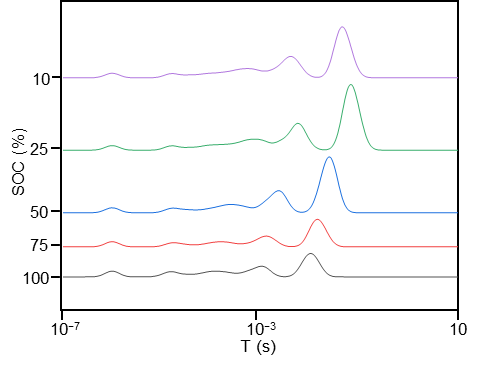
**

**Figure S14.** *In-situ* DRT time constant distribution spectra of the S@blank||Zn battery at different SOCs.

**
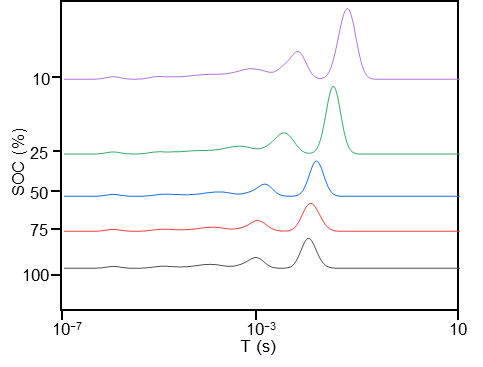
**

**Figure S15.** *In-situ* DRT time constant distribution spectra of the S@MES||Zn battery at different SOCs.


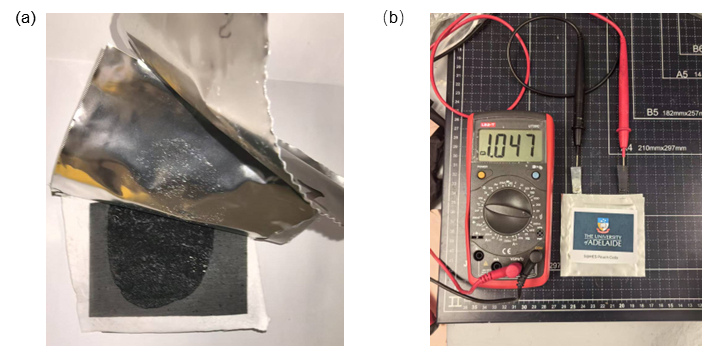


**Figure S16.** (a) Photograph of dissembled PC-HES. (b) Open circuit voltage of PC-HES.

**
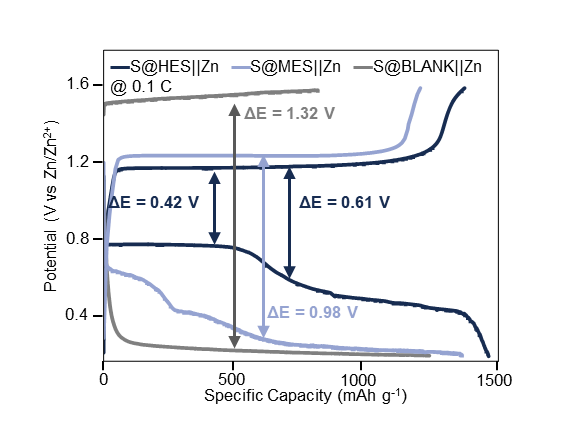
**

**Figure S17.** Polarization profiles of coin-type batteries with and without catalysts.

**
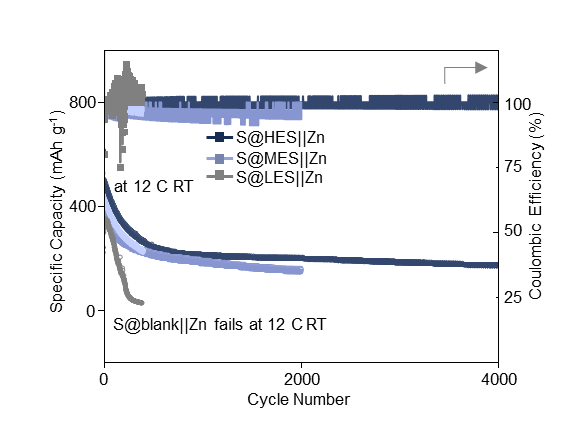
**

**Figure S18.** Fast-charging test for coin-type batteries at 12 C (20 A g^−1^).

**
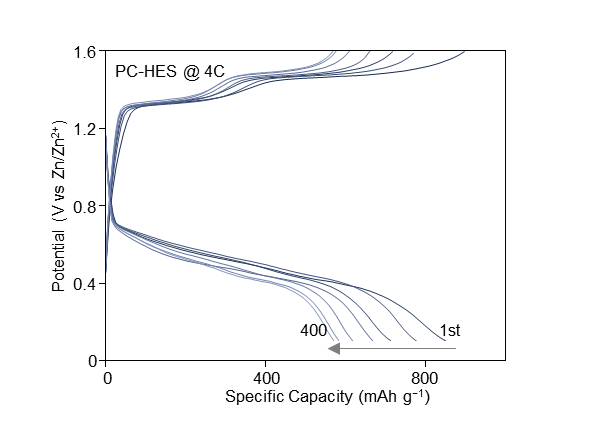
**

**Figure S19.** GCD profiles of PC-HES at 4 C from 1^st^ to 400^th^ cycles.

**
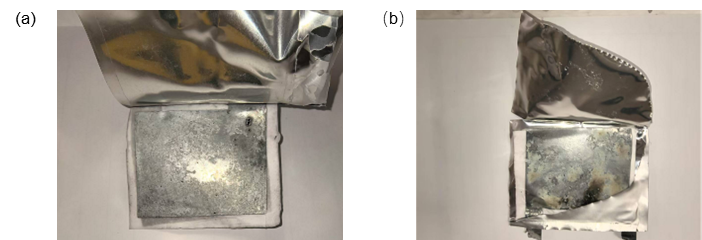
**

**Figure S20.** Corrision evoluation of (a) PC-HES and conventional (b) iodine-based pouch AZSBs after low-rate cycling.


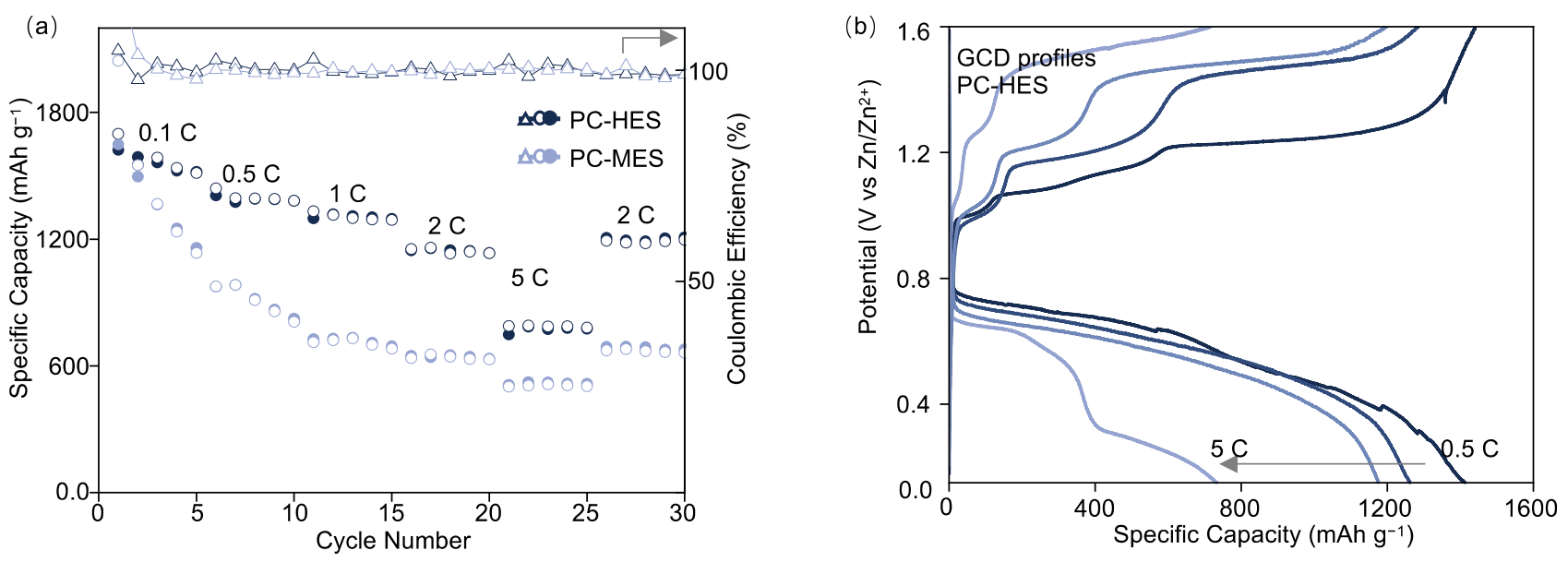


**Figure S21.** (a) Rate performance of PC-HES and PC-MES with (b) GCD profiles of PC-HES at 0.5, 1, 2, and 5C.

**Table S1.** ICP-MS data of the elemental composition ratios in the initial HES.

| Sample | Elements content (wt. %) | | | | | | | Entropy | |
| --- | --- | --- | --- | --- | --- | --- | --- | --- | --- |
|  | Fe | Co | Mn | Ni | Cu | Zn | S | ΔS_Full_ | ΔS_ES_ |
| HES | 8.84 | 16.32 | 8.16 | 16.88 | 17.68 | 0 | 32.12 | 1.53 | **1.56** |

**Table S2.** Changes of Composition and configurational entropy of electrode powders for S@HES||Zn and S@MES||Zn batteries before and after cycling (charged state).

| Sample | Elements content (wt. %) | | | | |  |  | Entropy | **Entropy** |
| --- | --- | --- | --- | --- | --- | --- | --- | --- | --- |
|  | Fe | Co | Mn | Ni | Cu | Zn | S | S_Full_ (R) | **S_ES_ (R)** |
| Initial HES | 8.84 | 16.32 | 8.16 | 16.88 | 17.68 | 0 | 32.12 | 1.53 | **1.56** |
| 2nd HES | 8.32 | 15.52 | 8.01 | 15.6 | 17.44 | 3.02 | 32.09 | 1.59 | **1.56** |
| 10^th^ HES | 8.21 | 15.33 | 7.94 | 15.44 | 17.82 | 3.81 | 31.45 | 1.6 | **1.56** |
| Initial MES | N.A. | 28.44 | 17.33 | 25.32 | N.A. | 0 | 28.91 | 1.31 | **1.08** |
| 2nd MES | N.A. | 24.23 | 15.27 | 22.51 | N.A. | 8.66 | 29.33 | 1.54 | **1.08** |
| 10^th^ MES | N.A. | 22.11 | 8.22 | 18.98 | N.A. | 14.54 | 36.15 | 1.34 | **1.03** |

**Table S3.** Performance comparison between S@HES||Zn batteries and reported AZSBs.

| Cathode  in Electrolyte | Catalyst  /RM | S_loading_  (mg cm^−2^) | ED_Cathode_  (Wh kg^−1^@ C) | Mean plateau  (V@ C) | Capacity loss per cyler (mAh g^−1^@C\|\|cycles) |
| --- | --- | --- | --- | --- | --- |
| S in ZnOAc^[1]^ | I_2_ | 0.7 | 42@0.06 | 0.4@0.06 | 25@0.06\|\|2 |
| S@S,N−CNF in ZnSO_4_^[2]^ | ZnI_2_ | 1-2 | 21@0.06 | ~0.56@0.06 | 7.8@0.06\|\|100 |
| ZnS^[3]^ | TU | 3.33_S-based_ | 198@0.06 | ~0.52@0.06 | 17@0.06\|\|35 |
| S in ZnSO_4_^[4]^ | ZnI_2_ | 2-2.5 | 222@0.1 | ~0.61@0.1 | 13@0.1\|\|40 |
| S in ZnOTF^[5]^ | G4-I_2_ | 1.2-1.6 | 135@0.06 | ~0.38@0.06 | 5.57@1\|\|130 |
| SeS7 in ZnOTF^[6]^ | G4-I_2_ | 1.2-1.4 | 208@0.03 | ~0.41@0.03 | 1.2@1.2\|\|250 |
| S/NC-CoO in ZnSO_4_^[7]^ | NA | 2 | 201@0.01 | ~0.54@0.01 | 3.2@2\|\|200 |
| S in ZnOTF^[8]^ | G4-I_2_ | 10 | 60@0.06 | 0.47@0.06 | 6@1.2\|\|50 |
| ZnS_0.93_Se_0.07_ in ZnSO_4_^[9]^ | ZnI_2_ | 3.2 | 285@0.06 | ~0.58@0.06 | 3.7@0.2\|\|30 |
| S in ZnSO4 | HES | 5 | **313@0.1** | **0.61@0.15** | **0.5@4\|\|400** |

**Reference**

[1] W. Li, K. Wang, K. Jiang, *Adv. Sci.* **2020**, *7*, 2000761.

[2] J. Li, J. Liu, F. Xie, R. Bi, L. Zhang, *Angew. Chem. Int. Ed.* **2024**, 63, e202406126.

[3] J. Li, J. Cong, Y. Ren, H. Ji, Z. Li, Y. Huang, *Energy Storage Mater.* **2024**, *70*, 103541.

[4] J. Li, Z. Cheng, Z. Li, Y. Huang, *Mater. Horiz.* **2023**, *10*, 2436-2444.

[5] M. Yang, Z. Yan, J. Xiao, W. Xin, L. Zhang, H. Peng, Y. Geng, J. Li, Y. Wang, L. Liu, Z. Zhu, *Angew. Chem. Int. Ed.* **2022**, *61*, e202212666.

[6] H. Zhang, M. Yang, J. Xiao, Z. Wu, W. Xin, X. Xiao, M. Niu, Z. Yan, Z. Zhu, *Adv. Funct. Mater.* **2024**, *34*, 2406125.

[7] M. Wang, H. Zhang, T. Ding, F. Wu, L. Fu, B. Song, P. Cao, K. Lu, *Sci. China Chem.* **2024**, *67*, 1531-1538.

[8] M. Yang, Z. Yan, H. Zhang, J. Li, Z. Zhu, L. Liu, L. Jiao, *Adv. Funct. Mater.* **2024**, *34*, 2406077.

[9] Y. Ren, J. Li, Y. Zhang, Y. Huang, Z. Li, *Small* **2024**, *20*, 2402466.
